# Trio and CRMP2 regulate axon branching and Semaphorin3A signaling

**DOI:** 10.1038/s42003-025-08988-8

**Published:** 2025-11-25

**Authors:** Erin Fingleton, Alexandra Lombardo, Sehoon Won, Kai Chang, Yan Li, Katherine W. Roche

**Affiliations:** 1 Receptor Biology Section, National Institute of Neurological Disorders and Stroke (NINDS), NIH, Bethesda, MD USA; 2 Department of Neuroscience, Brown University, Providence, RI USA; 3 Protein/Peptide Sequencing Facility, NINDS, NIH, Bethesda, MD USA

**Keywords:** Cellular neuroscience, Molecular neuroscience

## Abstract

Trio is a neuronally expressed, Rac1- and RhoA-activating RhoGEF, that is required for neurodevelopment. Mutations affecting the Rac1-activating GEF domain of Trio are associated with profound neurodevelopmental delay and Trio knock-out is embryonic lethal. Although there are studies showing a role for Trio in axon patterning, our understanding of the mechanistic underpinnings of Trio function is incomplete. We have now taken an unbiased approach to identifying the interactome of Trio in embryonic axonal compartments. Using immunoprecipitation-mass spectrometry, we identified the Collapsin Response Mediator Protein 2 (CRMP2) as a robust association partner of growth cone-localized Trio. Like Trio, CRMP2 has a well-known role in shaping the cytoskeleton, particularly during axon patterning. In the current study, we demonstrate Trio preferentially interacts with phosphorylated CRMP2 (pCRMP2) and is recruited by pCRMP2 to limit filopodial motility and axon branching. By introducing a GEF1-ablating disease-related mutation, we further demonstrate that Trio-GEF1 signaling is required for pCRMP2-mediated axon branch suppression. Finally, we show that Semaphorin3A invokes pCRMP2-Trio signaling to limit axon branching in vitro, revealing a developmental role for pCRMP2-Trio signaling.

## Abstract (web summary)

Unbiased mass spectrometry analysis reveals a phosphorylation-dependent CRMP2-Trio interaction that limits axon branching downstream of Sema3A.

## Introduction

Accurate axon pathfinding and target innervation are essential to the development of the nervous system. Guided by growth cones, axons traverse long distances and bypass inappropriate connections to reach appropriate anatomical targets. Axons may then form branches to adequately innervate the target population, then prune these branches to form precise and sparse connections^1,2^. Axon patterning is actualized through the dynamic reorganization of the cytoskeleton in response to extracellular cues and intracellular growth programs^3–5^. Dysregulation of these pathways is genetically associated with neurodevelopmental disorders (NDDs), and white matter differences are observed in NDDs^6–9^.

Trio is a RhoGEF that activates Rac1 and RhoA, small GTPases that remodel the actin cytoskeleton and are essential for axon patterning^5,10^. Trio is highly implicated in NDDs, specifically through de novo mutations affecting the Rac1-activating GEF domain (GEF1)^11–13^. NDD-related mutations in Trio can either cause severe developmental delay and macrocephaly through constitutive GEF1 activation, or moderate developmental delay and microcephaly through GEF1 loss-of-function^14–22^. These observations suggest that Trio-mediated Rac1 activation is particularly critical for neurodevelopment. This is supported by Trio knockout (KO) and conditional Trio KO mouse models, which exhibit decreased survival rates, abnormal gross anatomy, and striking differences in white matter organization^23–25^. Additionally, explants and cultured neurons from Trio KO mice exhibit attenuated responses to both attractive and repulsive axon guidance molecules^24–26^. While Trio clearly mediates some forms of axon patterning, the question of how Trio GEF function is invoked in these pathways is still outstanding.

Using an unbiased proteomic approach, we identified Collapsin Response Mediator Proteins (CRMPs) as Trio-interacting partners in the growth cone. In particular, we examined the association of Trio with CRMP2, because CRMP2 is more broadly expressed than other CRMPs and more strongly linked genetically to NDDs than other CRMPs^27–29^. CRMP2 is a bidirectional regulator of axon morphology and is heavily regulated by COOH-terminal phosphorylation^30,31^. Non-phosphorylated CRMP2 promotes axon outgrowth and branching, whereas phosphorylated CRMP2 (pCRMP2) mediates axon repulsion and branch pruning downstream of semaphorins, a class of repulsive axon guidance cues^31–35^.

Here, we elucidate the functional consequences of the association between Trio and CRMP2. We characterize a selective association between Trio and pCRMP2, which is dependent on CRMP2 phosphorylation. Additionally, we demonstrate pCRMP2 requires the Rac1-GEF of Trio to limit filopodial motility and axon branching, and that this pathway is compromised by an NDD-related mutation in Trio. Finally, we show that the pCRMP2-Trio association is invoked by and required for Semaphorin3A-induced axon repulsion.

## Results

### Identification of pCRMP2 as a Trio interacting protein

**Fig. 1:**
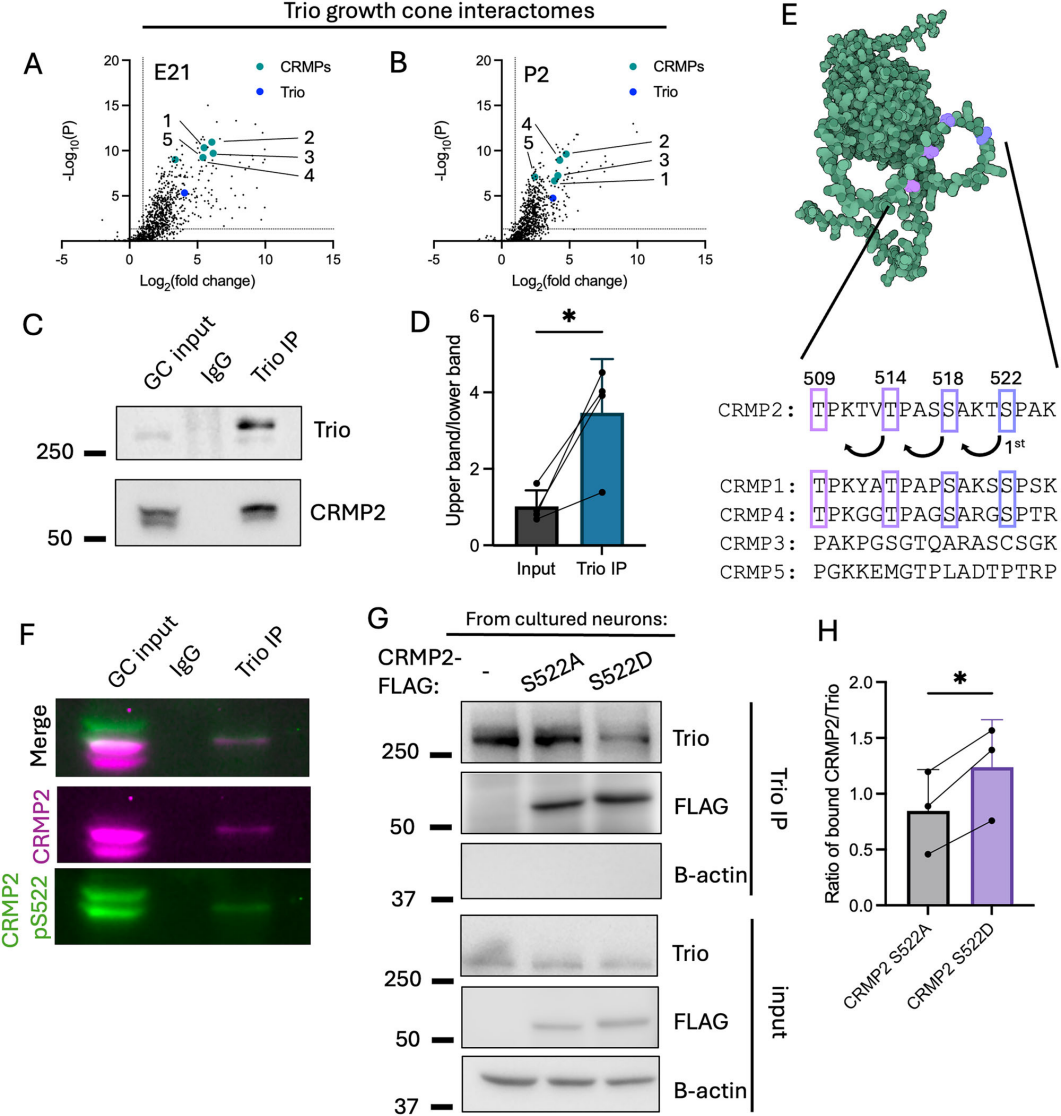
pCRMP2 and Trio interact in the growth cone (GC) fraction. **A**, **B** Trio was immunoprecipitated from growth cone fractions collected at embryonic day 21 (E21) (**A**) and postnatal day 2 (P2) (**B**) and subjected to mass spectrometry (N = 3 litters), with results depicted as volcano graphs and CRMPs 1-5 labeled with their corresponding numbers. **C** Immunoprecipitation of Trio from embryonic day 18 growth cone fraction. **D** Quantification of CRMP2 upper/lower band ratio in (**C**) (N = 3 litters). Data are represented as paired means ± SEM (ratio paired two-sided t-test, p = 0.0149). **E** Alphafold-predicted structure of human CRMP2 (DPYSL2) with C-terminal phosphosites highlighted with alignment of phosphosite-bearing region across CRMP family members. **F** Immunoblot probing with CRMP2 pS522 antibody. **G** Immunoprecipitation of endogenous Trio from primary cortical culture transfected with CRMP2-FLAG S522A or S522D. **H** Quantification of (**G**) (N = 3 experimental replicates, Ratio paired t-test: p = 0.0282).

Previously, we identified the family of CRMPs in a complex with Trio isolated from the adult rat synaptosomal fraction^36^. CRMP2 is a key mediator of growth cone dynamics, so we hypothesized Trio and CRMP2 may interact in the growth cone at earlier developmental timepoints. To investigate earlier stages of development, we isolated a growth-cone enriched fraction from rats at embryonic day 21 (E21) and postnatal day 2 (P2), immunoprecipitated Trio, and performed mass-spectrometry on the Trio-immunoprecipitated complexes (Fig. 1A, B, Supplementary Data 1). At both time points, we observed an association between Trio and CRMP1-5. To learn more, we immunoprecipitated Trio from the growth cone fraction, resolved the immunoprecipitated sample by SDS-PAGE, and immunoblotted for Trio and CRMP2 (Fig. 1C). In the growth cone fraction, we observed two CRMP2 bands, whereas in the Trio-immunoprecipitate (Trio IP) we observe a preferential enrichment of the upper CRMP2 band (Fig. 1C, D). To more precisely dissect the Trio-CRMP2 association, we co-expressed Trio and CRMPs 1-5 in HEK293T cells and performed co-immunoprecipitation experiments (Fig. S1). We were unable to co-immunoprecipitate Trio with any CRMP, indicating this association may be indirect.

CRMP2 undergoes well-characterized C-terminal phosphorylation on T509, T514, S518, and S522, which results in an observed mobility shift on an immunoblot (Fig. 1E)^31,32,37^. As such, we immunoblotted for CRMP2 pS522 and observed that Trio interacts with C-terminally phosphorylated CRMP2 (pCRMP2) (Fig. 1F). To assess whether CRMP2 phosphorylation is required for this association, we immunoprecipitated Trio, performed on-bead dephosphorylation, resolved by SDS-PAGE, and then immunoblotted for Trio and CRMP2 (Fig. S2). We observe a significant decrease in co-immunoprecipitated CRMP2 after dephosphorylation (Fig. S2). C-terminal CRMP2 phosphorylation is primed by Cdk5-mediated phosphorylation on CRMP2 S522, after which T509, T514, and S518 are phosphorylated by GSK3B (Fig. 1E)^32,33,37^. We reasoned we could study these phosphorylation events in an all-or-none fashion by mutating only S522 to an alanine (S522A) to block C-terminal phosphorylation of CRMP2, or an aspartic acid (S522D) to mimic C-terminal phosphorylation of CRMP2. To check whether Trio interacts with our phospho-mutant CRMP2, we transiently transfected primary cortical neurons with CRMP2-FLAG S522A or S522D and immunoprecipitated Trio. Via immunoblotting, we find that Trio binds more robustly to CRMP2 S522D than CRMP2 S522A (Fig. 1G, H). pCRMP2 exerts suppressive effects on axon morphology: overexpression of phosphomimetic CRMP2 limits axon branching and pCRMP2 is required for growth cone collapse and axon pruning downstream of Semaphorin3A (Sema3A), a well-known repulsive axon guidance cue. The observed preferential association between pCRMP2 and Trio suggest a non-canonical role for Trio in limiting growth cone motility and axon branching.

### pCRMP2-Trio suppresses filopodial motility

**Fig. 2:**
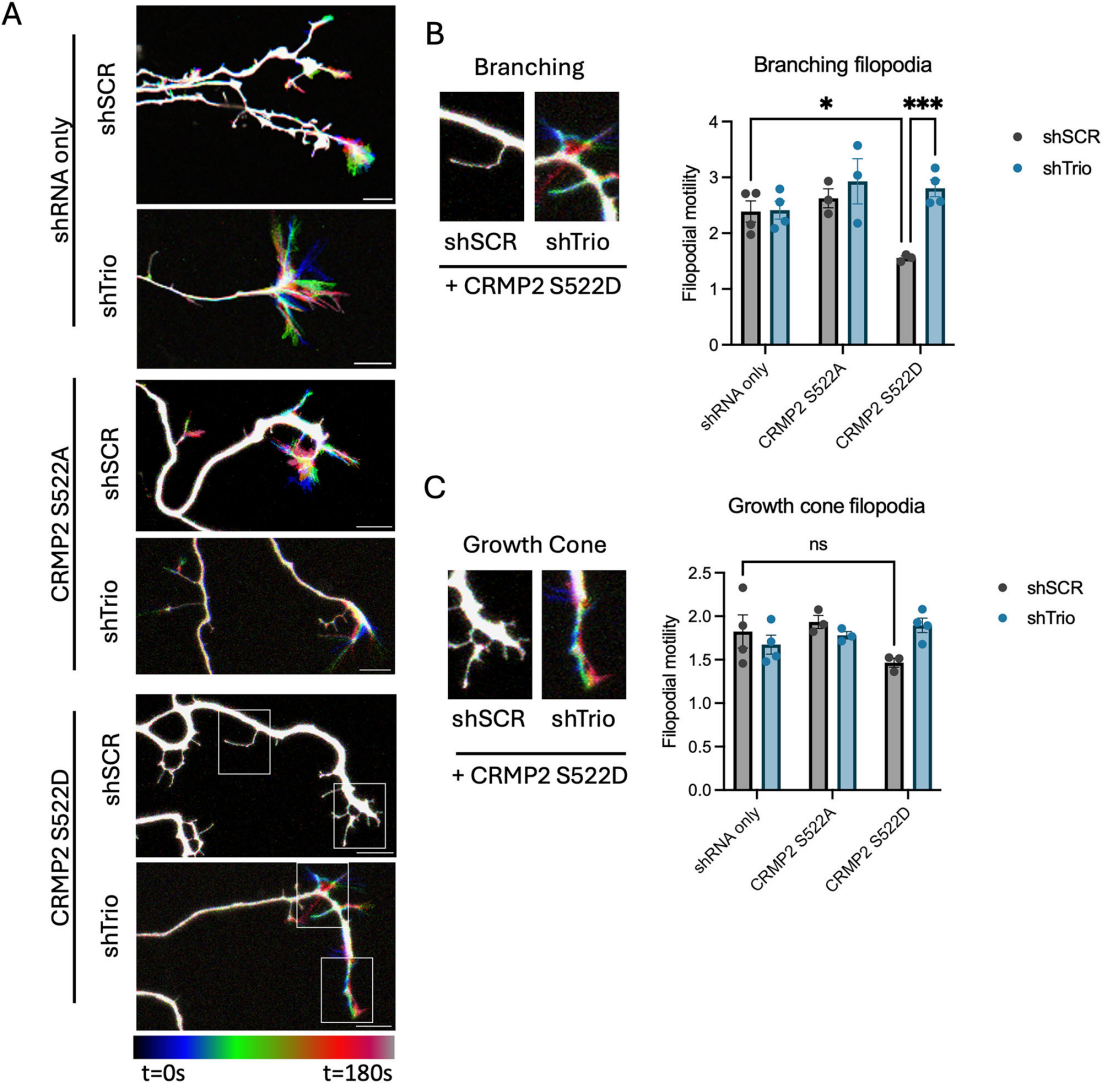
pCRMP2 and Trio suppress filopodial motility. **A** DIV 3 neurons were transfected with shSCR-GFP or shTrio-GFP and SNAP-CRMP2 S522A or S522D, and labeled with SNAP-Cell TMR-STAR and imaged at DIV 7 for 6 minutes. Representative images are temporally-coded t-stack projections. N = 4 experimental replicates, n = 5–8 neurons. **B** Branching filopodial motility was measured by taking a ratio of time projected filopodial area to single-time point filopodial area. Two way ANOVA reveals significant interaction (p = 0.0210), CRMP2 expression effect (p = 0.0409), and shRNA expression effect (p = 0.0069). Post-hoc Sidak’s multiple comparisons indicate significant differences between shSCR + CRMP2 S522D and shTrio + CRMP2 S522D (p = 0.0007), but not shSCR vs shTrio or shSCR (p = 0.9293) + CRMP2 S522A vs shTrio + CRMP2 S522A (p = 0.3440), as well as differences between shSCR and shSCR + CRMP2 S522D (p = 0.0355) and shSCR + CRMP2 S522A and shSCR + CRMP2 S522D (p = 0.0110), but not shSCR and shSCR + CRMP2 S522A (p = 0.8131). No differences were observed between shTrio and shTrio + CRMP2 S522A or S522D (p = 0.2616, 0.4224, respectively) or shTrio + CRMP2 S522A and shTrio + CRMP2 S522D (p = 0.9648). **C** Growth cone filopodial motility was measured by taking a ratio of time projected filopodial area to single time point filopodial area. Two way ANOVA reveals significant interaction effect (p = 0.0393), but no effect of shRNA expression (p = 0.6767) or CRMP2 expression (p = 0.3710). Post-hoc Sidak’s multiple comparisons indicate a significant difference in shSCR vs shTrio in CRMP2 S522D expressing cells (p = 0.0217), but no significant differences in shSCR vs shTrio expressing cells in shRNA alone (p = 0.3438) or CRMP2 S522A (0.4035).

Whereas growth cones typically feature highly motile filopodia that survey the environment for cues, Sema3A-exposed growth cones collapse and become “paralyzed,” featuring limited filopodial activity^38^. Since pCRMP2 is required for Sema3A-induced repulsion and Trio regulates the actin cytoskeleton through its GEF domains, we reasoned co-expression of CRMP2 S522D and knock-down of *Trio* might alter filopodial dynamics. We transiently transfected primary hippocampal neurons with constructs that co-express short hairpin RNA (a control or scramble or against *TRIO*) and GFP under separate promoters (shSCR or shTrio), and SNAP-CRMP2 S522A or S522D at days in vitro 3 (*DIV 3*)^39^. We labeled neurons with SNAP-Cell TMR-STAR and performed live cell imaging at *DIV 7*. We observe that CRMP2 S522D-expressing filopodia show limited motility compared to CRMP2 S522A-expressing filopodia (Figs. 2A, B, S3). *Trio* knock-down (shTrio) restores filopodial motility in CRMP2 S522D-expressing neurons to levels comparable to CRMP2 S522A-expressing neurons (Fig. 2A, B). We observed these altered filopodia dynamics in filopodia distant from the axon tip, which might ultimately form axon branches (Fig. 2B, “branching”). In filopodia near the axon tip, which might constitute sensory elements of the extending axon (Fig. 2C, “Growth Cone”), though we observed a similar trend, CRMP2 S522D expression did not suppress filopodial motility compared to shRNA alone.

### pCRMP2-Trio limits axon branching in vitro

**Fig. 3:**
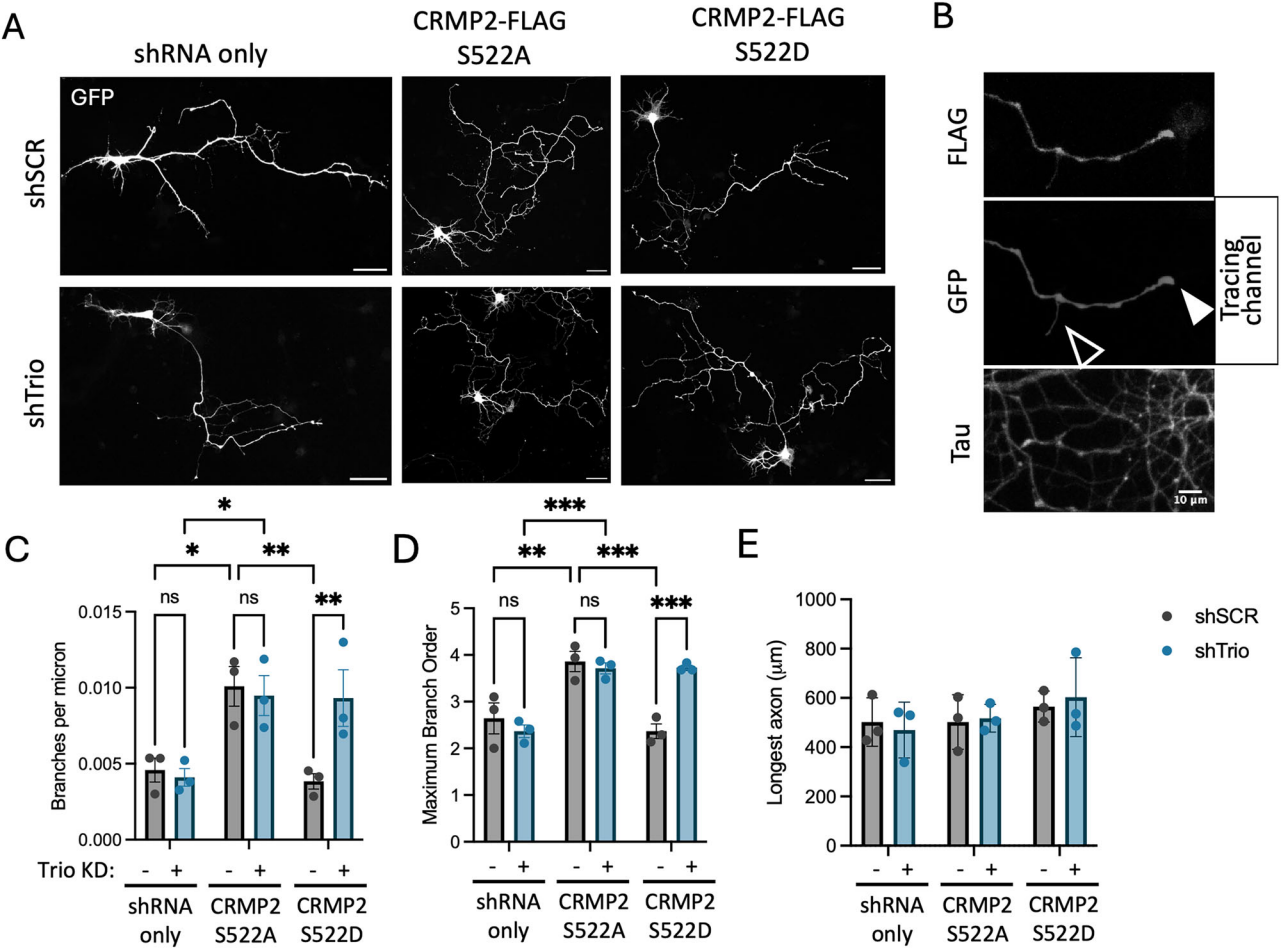
pCRMP2 and Trio limit axon branching. **A** DIV 3 neurons were transfected with shSCR-GFP or shTrio-GFP and CRMP2-FLAG S522A or S522D, and stained against FLAG, GFP, and tau at DIV 7. Tau positive neurites (axons) were traced and measured using Simple Neurite Tracer. (N = 3 experimental replicates, n = 7-10 neurons) Scale bar = 50 microns. **B** Example of traced (filled arrow) and non-traced branch (empty arrow). **C** Axon branch density was measured. Two-way ANOVA revealed a significant CRMP2 expression effect (p = 0.0019) and interaction effect (p = 0.0352), but no shRNA effect (p = 0.1485). Post-hoc Tukey’s tests reveal a significant different between shRNA only and CRMP2 S522A-expressing neurons in shSCR and shTrio conditions (p = 0.0146 and p = 0.0169, respectively), no significant difference between shRNA only and CRMP2 S522D expressing neurons in shSCR conditions (p = 0.8960), a significant difference between shSCR and shTrio in CRMP2 S522D (p = 0.0059), but not in shRNA only (0.7766) or CRMP2 S522A conditions (0.7169). **D** Maximum branch order was measured. Two-way ANOVA revealed a CRMP2 expression effect (p = 0.0016) and interaction effect (p < 0.0001), but not a significant shRNA effect (p = 0.0666). Post-hoc comparisons reveal significant differences between shRNA only and CRMP2 S522A expressing neurons in shSCR and shTrio conditions (p = 0.0019 and 0.0008, respectively), no significant difference between shRNA only and CRMP2 S522D neurons in shSCR conditions (p = 0.5740), and significant differences between shSCR and shTrio in CRMP2 S522D (p = 0.0003), but not shRNA only (p = 0.3241) or CRMP2 S522A conditions (p = 0.5957). **E** Longest axon was measured using Simple Neurite Tracer longest shortest path measurement. Two-way ANOVA reveals no statistically significant differences (interaction: p = 0.8654; shRNA condition: p = 0.9252; CRMP2 overexpression: p = 0.2843).

Axonal filopodia may mature into axon branches, thus we hypothesized that influence of pCRMP2 and Trio on filopodial dynamics may track with alterations to axon branching. Indeed, CRMP2 plays a well-known role in regulating axon branching in a phosphorylation-dependent manner^40^. To that end, we transiently transfected primary hippocampal neurons with shSCR-GFP or shTrio-GFP and CRMP2-FLAG WT, S522A or S522D at *DIV 3*. Neurons were fixed at *DIV 7* and stained against GFP, FLAG, and Tau (Figs. 3A, B, S4, S5). Using Simple Neurite Tracer, we traced tau-positive axons and measured axon length and branching (Fig. 3C–E)^41^. Consistent with previous reports, we find that over-expression of WT CRMP2 or CRMP2 S522A results in increased axon branching, whereas over-expression of CRMP2 S522D does not (Figs. 3A, C, S5)^31^. Trio knock-down does not suppress CRMP2 S522A-induced axon branching, consistent with our findings that Trio preferentially interacts with pCRMP2. However, we find that Trio knockdown relieves the null effect of overexpressing CRMP2 S522D, resulting in significantly increased axon branching compared to shSCR + CRMP2 S522D expressing neurons. We hypothesize that pCRMP2 recruits Trio to locally auto-inhibit CRMP2-mediated axon branching, such that Trio knockdown permits CRMP2-mediated axon branching despite CRMP2 phosphorylation.

### pCRMP2 signaling depends on the Rac1-GEF of Trio

**Fig. 4:**
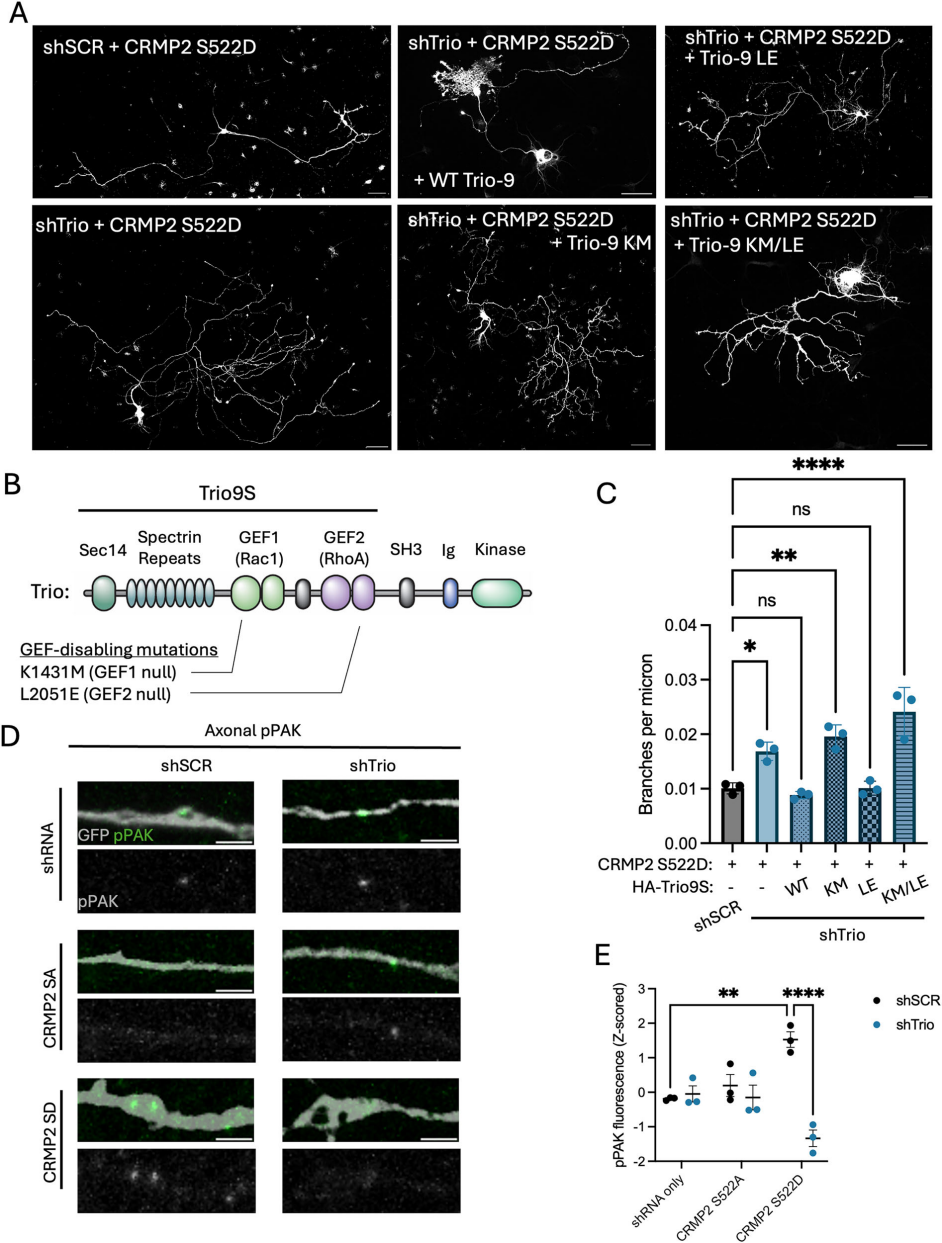
pCRMP2 and Trio regulate branching via the GEF1 domain. **A** DIV 3 neurons were transfected with shSCR-GFP or shTrio-GFP, CRMP2-FLAG S522D and HA-Trio9S WT, K1431M (KM), L2051E (LE) or K1431M/L2051E (KM/LE), and stained against HA and GFP at DIV 7. Presumptive axons were traced and measured using Simple Neurite Tracer. (N = 3 experimental replicates, n = 7-10 neurons). Scale bar = 50 microns. **B** Schematic of full-length Trio domains with Trio9S splice isoform highlighted. GEF-disabling mutations are identified. **C** Quantification of (**A**). Data are represented as means ± SEM (N = 3). ANOVA revealed significant differences between groups (p < 0.0001). Post-hoc Dunnett’s multiple comparisons tests revealed significant differences between shSCR + CRMP2 S522D and shTrio + CRMP2 S522D (p = 0.0123), shTrio + CRMP2 S522D + HA-Trio9S K1413M (p = 0.0010), and shTrio + CRMP2 S522D + HA-Trio9S K1431M/L2051E (p < 0.0001), but not between shSCR + CRMP2 S522D and other groups (WT: p = 0.9379, LE: p = >0.9999). **D** DIV 3 neurons were transfected with shSCR-GFP or shTrio-GFP, CRMP2-FLAG S522A or CRMP2-FLAG S522D and stained against GFP, FLAG, and pPAK at DIV 7. Axonal pPAK intensity was analyzed in FIJI (N = 3 experimental replicates, n = 7–10 neurons). Representative image brightness and contrast were adjusted to enhance clarity of the pPAK signal and its overlap with the axon shaft. Image adjustments were applied equally across conditions. **E** Quantification of (**D**). Data are represented as z-scored means ± SEM. Two-way ANOVA reveals a significant interaction (p = 0.0002) and CRMP2 expression effect (p = 0.0004), but not a significant shRNA effect (p = 0.7027). Multiple post-hoc tests reveal significant differences between shRNA only and CRMP2 S522D expressing neurons in shSCR and shTrio conditions (p = 0.0013 and 0.0103, respectively) and shSCR + CRMP2 S522D and shTrio + CRMP2 S522D (p < 0.0001).

We next tested if pCRMP2 signaling is mediated by one of Trio’s GEF domains, which can activate either Rac1 or RhoA. To identify which, if either, of Trio’s GEF domains may function downstream of pCRMP2, we performed rescue experiments with HA-tagged shRNA-resistant Trio-9S (the dominant Trio splice isoform in the brain), harboring GEF-altering mutations (Fig. 4A, B). To block GEF1 function, we introduced a K1431M mutation, which is NDD-associated, ablates GEF1 function, and phenocopies other NDD-associated GEF1 mutations^17,22^. To block GEF2 function, we introduced an L2051E mutation, which was previously demonstrated to ablate GEF2 function^42,43^. We find that wild-type Trio-9S (WT) and GEF2 loss-of-function Trio-9S (L2051E) can rescue branch suppression downstream of CRMP2 S522D, but not GEF1 loss-of-function Trio-9S (K1431M) or GEF1/2 loss-of-function Trio-9S (K1431M/L2051E) (Fig. 4C). Expression of Trio GEF mutants in absence of CRMP2 S522D does not exert a significant effect on axon branching (Fig. S6). The GEF1 domain of Trio activates Rac1, resulting in phosphorylation of PAK1 (pPAK1), the immediate downstream effector of Rac1. We measured pPAK signal in the axon shafts of primary hippocampal neurons expressing shSCR-GFP or shTrio-GFP and CRMP2 S522A or CRMP2 S522D (Fig. 4D, E). We find that CRMP2 S522D drives an increase in axonal pPAK fluorescence in a Trio-dependent manner, consistent with a role for pCRMP2 in driving Rac1 activation through the GEF1 domain of Trio.

### pCRMP2-Trio signaling is invoked by Sema3a

**Fig. 5:**
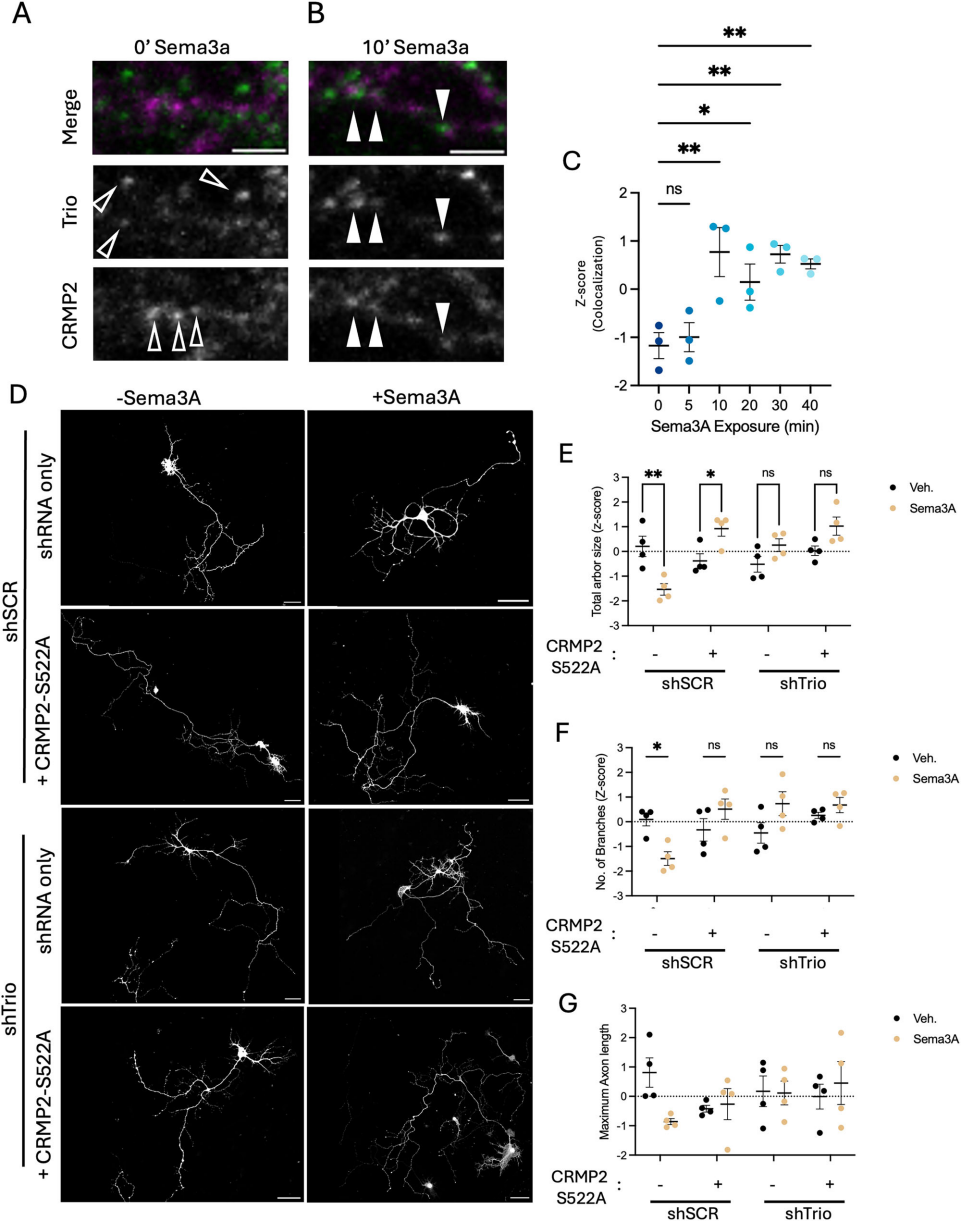
CRMP2 and Trio mediate Semaphorin3A signaling. **A** DIV 3 neurons were transfected with GFP and then stained against Trio, CRMP2, and GFP. In merged image, Trio stain is shown in green, CRMP2 stain is shown in magenta. **B** DIV 3 neurons were transfected with GFP and treated with Sema3A (700 ng/ml) for 10 minutes, then stained against Trio, CRMP2, and GFP. In merged image, Trio stain is shown in green, CRMP2 stain is shown in magenta. **C** DIV 3 neurons were transfected with GFP and treated with Sema3A (700 ng/ml) for the indicated times and then stained against Trio, CRMP2, and GFP (N = 3 experimental replicates, n = 7–10 axon compartments). Trio and CRMP2 colocalization was measured within GFP+ axons and is represented as z-scored means means ± SEM. One-way ANOVA reveals a significant effect (p = 0.0006). Multiple Holm-Sidak’s comparisons indicate significant differences between Trio-CRMP2 colocalization at baseline (0 minutes) compared to 10 minutes (p = 0.005), 20 minutes (p = 0.0251), 30 minutes (p = 0.005), and 40 minutes (p = 0.0080) but not 5 minutes (p = 0.7040). Representative image brightness and contrast were adjusted to enhance the clarity of the Trio and CRMP2 signals and their overlap. Image adjustments were applied equally across conditions. **D** DIV 3 neurons were transfected with shSCR/shTrio-GFP and CRMP2-FLAG S522A (SA), then treated with 700 ng/ml Sema3A for 72 hours and stained for GFP, FLAG, and Tau (GFP shown here). Tau positive neurites (axons) were traced and measured using Simple Neurite Tracer. (N = 4 experimental replicates, n = 7-10 neurons). **E** Total axon arbor length was measured using Simple Neurite Tracer and is represented as z-scored means ± SEM. Two-way ANOVA reveals a significant interaction effect (p = 0.0001) and transfection condition effect (p = 0.0038), but no Sema3A treatment effect (0.1340). Multiple post-hoc tests reveal a significant difference in total axon arbor length between Sema3A-treated and vehicle-treated conditions in shSCR-expressing neurons (p = 0.0019) and shTrio-expressing neurons (p = 0.0228), but no other conditions (shSCR + CRMP2 S522A, p = 0.2973; shTrio + CRMP2 S522A, p = 0.1120). **F** Branch number was measured using Simple Neurite Tracer and represented as z-scored means ± SEM. Two-way ANOVA reveals a significant interaction effect (p = 0.0037) and transfection condition effect (p = 0.00241), but no Sema3A treatment effect (0.4098). Multiple post-hoc Sidak’s tests reveal a significant difference in branch number between Sema3A-treated and vehicle-treated conditions in shSCR-expressing neurons (p = 0.0194) but no other conditions (shSCR + CRMP2 S522A, p = 0.3868; shTrio, p = 0.1117; shTrio + CRMP2 S522A, p = 0.8856). **G** Maximum axon length was measured using Simple Neurite Tracer and is represented as z-scored means ± SEM. Two-way ANOVA reveals no significant interaction effect (p = 0.1226), transfection condition effect (p = 0.6278), or Sema3A treatment effect (0.4033).

Sema3A is a well-known repulsive axon guidance cue that triggers growth cone collapse and axon branch pruning^44,45^. Sema3A-signaling stimulates and requires C-terminal phosphorylation of CRMP2 and Rac1 activation^33,46,47^. Trio interacts with pCRMP2 in the growth cone fraction, so we hypothesized that Sema3A might invoke the pCRMP2/Trio association. To assess whether Sema3A stimulates an association between Trio and CRMP2 in the axon shaft, we transfected *DIV 3* hippocampal neurons with pCAG-GFP and then treated with Sema3A (700 ng/ml) the following day, and fixed at various time points and stained against GFP, Trio, and CRMP2. At baseline, we observed minimal colocalization between Trio and CRMP2 (Fig. 5A, C). In the axon shaft, we observed a transient increase in colocalization of Trio and CRMP2 after 10 minutes of Sema3A exposure (Fig. 5B, C). We also observe closely apposed Trio and CRMP2 puncta, rather than a completely overlapping distribution. This may further indicate their interaction is mediated by additional binding partners.

Previous studies demonstrate that chronic exposure of Sema3A can induce axon shortening and suppress axon branching in cultured hippocampal neurons^44,45^. To test if Trio and CRMP2 may be required for sema3A-signaling in culture, we transiently transfected *DIV 3* primary hippocampal neurons with shSCR-GFP or shTrio-GFP and CRMP2-FLAG S522A. At *DIV 4*, we exposed these neurons to 700 ng/mL Sema3A, and 72 hours later stained against GFP, FLAG, and Tau (Fig. 5D). To measure Sema3A signaling, we traced axons and measured total axonal arbor size (the sum of all branches), number of branches, and maximum axon length (the longest path traced from soma to any axon teminal) (Fig. 5E). As expected, Sema3A treatment decreased the total axonal arbor length and branch number (Fig. 5E, F). As previously observed, we find that CRMP2 S522A overexpression ablates repulsive Sema3A signaling (Fig. 5E, F)^33^. Additionally, we find that Trio knock-down blocks repulsive Sema3A signaling (Fig. 5E, F). Importantly, we do not observe a summative effect of overexpressing CRMP2 S522A and Trio knock-down, indicating that Trio and pCRMP2 act in the same pathway downstream of Sema3A. Surprisingly, we do not observe an effect of Sema3A on maximum axon length (Fig. 5G).

## Discussion

In the current study, we have elucidated a suppressive role for Trio GEF1 function downstream of pCRMP2 and Sema3A. We found that Trio interacts preferentially with pCRMP2 versus CRMP2 and regulates axon branching downstream of pCRMP2, but not CRMP2. These observations extend to Sema3A-induced branch suppression, which requires pCRMP2-Trio signaling. Further, we directly tie the pCRMP2-Trio signaling to NDD pathophysiology by demonstrating that the NDD-associated *TRIO* K1431M variant causes inappropriate axon branching despite CRMP2 phosphorylation. Excitingly, our findings contribute new insights into NDD-etiology, CRMP2 signaling, cytoskeletal determinants of axon patterning, and Rho GTPase signaling.

Intriguingly, we do not find that *TRIO* knock-down via shRNA decreases axon length, in contrast to findings that axon length is shortened in neuronal cultures from *TRIO* KO mice. Whereas *TRIO* KO ablates Trio function from the moment of plating to the moment of experimentation, we knock-down *TRIO* beginning at DIV 3. Therefore, other early processes that contribute to axon elongation, i.e. establishment of polarity and axon formation, may be intact in our experimental paradigm but disrupted in *TRIO* KO neuronal cultures, explaining the apparent discrepancy. Additional work establishing a role for Trio in axon elongation has been performed in vivo, where neurons receive instructive cues and growth factors that guide directional elongation. By contrast, our experiments are performed in vitro, where neurons receive a limited amount of positive or negative cues. In the context of the field, our work shows that Trio may not guide baseline axon elongation or branching, but is instead recruited to pathways upon exposure to an exogenous cue. Indeed, *Trio* knockdown only elicits ectopic axon branching in the presence of CRMP2 S522D, and CRMP2 is phosphorylated in response to exogenous cues.

It is well documented that dysregulated Trio GEF1 signaling causes NDDs, as mutations in the Trio GEF1 are generally GEF1 loss-of-function mutations and cause moderate developmental delay and microcephaly^14,15,17,19,20^. Our findings identify a role for Trio GEF1 signaling in regulating axon branching downstream of Sema3A and demonstrate that an NDD-associated GEF1 loss-of-function mutation (K1431M) causes exuberant branching instead of branch suppression downstream of pCRMP2. Sema3A coordinates developmental axon pruning and cortical circuit refinement, therefore it is likely these processes are impacted by Trio GEF1 mutations in vivo^45^. This hypothesis is supported by the observation that expression of pathogenic Trio GEF1 mutants in zebrafish motor neurons stimulates ectopic axon branching^15^. Thus, our work adds context to Trio-NDD pathophysiology. In addition, pCRMP2 mediates signaling downstream of other Semaphorins, which also mediate branch pruning^35,45^. Thus, Trio GEF1 mutations may impact developmental pruning schemes beyond those coordinated by Sema3A. Further work is needed to determine the role of pCRMP2-Trio in vivo. Even so, our data add to the growing body of evidence positioning Semaphorin signaling as an important NDD-impacted pathway^48–51^.

The GEF1 domain of Trio is typically conceptualized as a Rac1-activating domain, although it can also activate RhoG^10^. Here, we present evidence that pCRMP2 activates Rac1 through Trio and requires Rac1 to limit branching. However, it is possible that RhoG may be activated by Trio upstream of Rac1, or that RhoG might be acting downstream of pCRMP2 and Trio in a parallel pathway. Canonically, Rac1 is considered an outgrowth-promoting Rho GTPase; however, different experimental manipulations in different systems have revealed that Rac1 acts bidirectionally in axon patterning^52,53^. Sema3a and Ephrin-A2 require Rac1 to stimulate growth cone collapse, and Trio-Rac1 signaling negatively regulates invadopodia, which are protrusive axonal structures^46,47,54^. This suppressive Rac1 signaling may be driven by preferential activation of LIMK downstream of Rac1, which can limit actin treadmilling, and thus limit outgrowth and branching^55,56^. In fact, phosphorylation of cofilin by LIMK is required for Sema3A-signaling in vitro and in vivo^57^.

It is worth noting that Rac1 activation as a downstream requirement for Sema3A signaling is somewhat controversial, since RhoA has also been implicated downstream of Sema3A and is more generally associated with axon retraction^58^. It is plausible that Rac1 and RhoA may coordinate cytoskeletal remodeling in different subcellular micro-domains downstream of Sema3A. For example, Rac1 might stimulate bulk endocytosis and focal adhesion disassembly, which are required for Sema3A signaling, and RhoA might stimulate actin arc compaction, which halts microtubule advance^59–61^. Our work adds evidence for an important Rac1-mediated downstream component of Sema3A signaling but does not controvert a role for RhoA. Additional experimentation to elucidate cytoskeletal targets of pCRMP2-Trio signaling may further contribute to the resolution of this question.

**Fig. 6:**
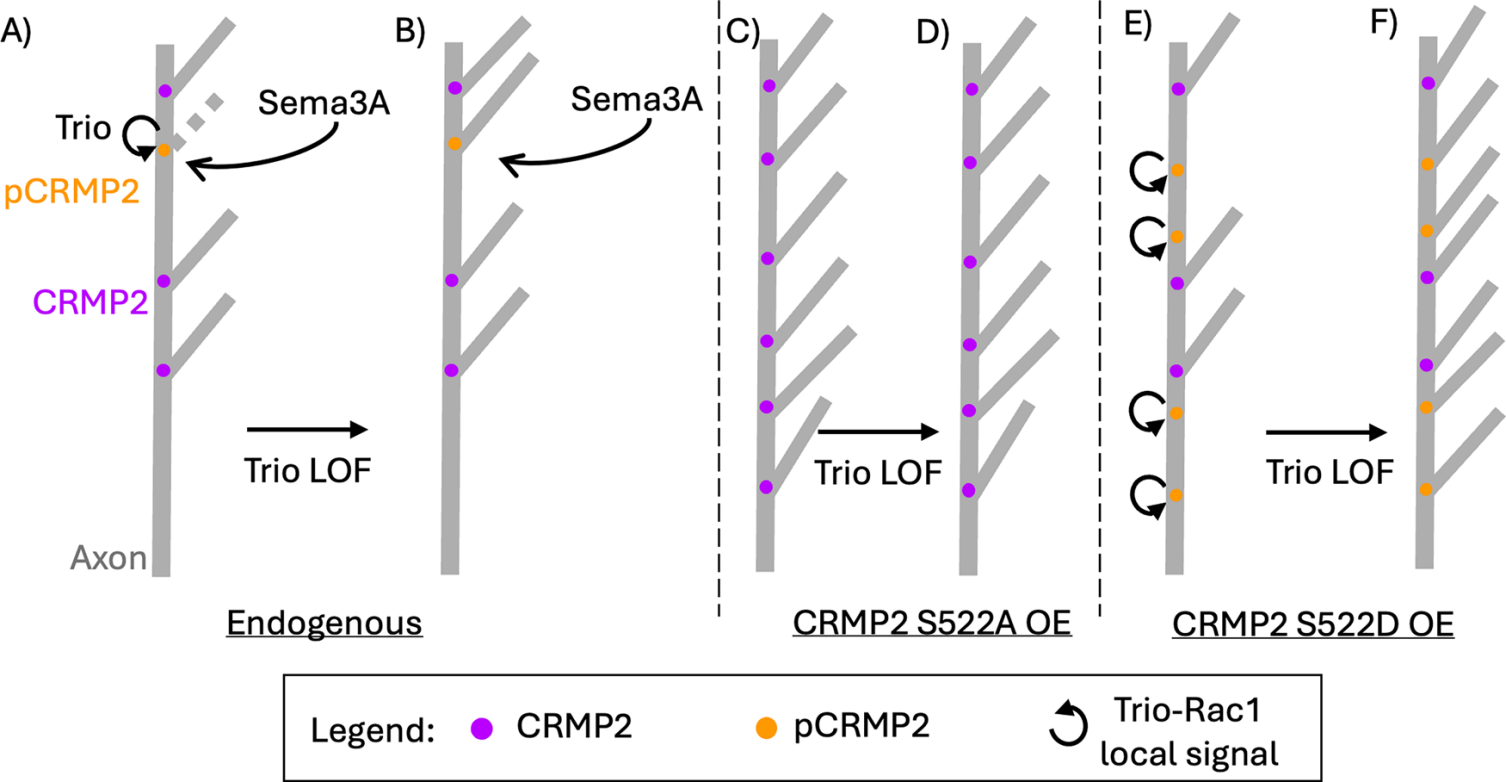
Model for CRMP2-mediated axon branching and its regulation by Trio. **A** CRMP2 foci mark branching zones along an axon. When CRMP2 is nonphosphorylated (purple circles) these zones are permissive and branches may form. When CRMP2 is phosphorylated (orange circles) these zones are non-permissive due to the recruitment of Trio. Sema3A (green gradient) can locally convert CRMP2 foci to pCRMP2 foci to limit axon branching in a Trio-dependent manner. **B** When Trio-Rac1 signaling is lost (either through mutation of GEF1 or Trio knock-down), sema3A can locally convert CRMP2 foci to pCRMP2 foci, but Trio is not recruited to limit axon branching. Thus, the axon branches despite sema3A application. **C** When CRMP2 S522A is overexpressed (OE), new ectopic branching zones are created. Because CRMP2 S522A does not recruit Trio, these new zones are permissive and branching increases. **D** Ectopic branching downstream of CRMP2 S522A overexpression (OE) is not changed by Trio loss-of-function (LOF) because CRMP2 S522A does not recruit Trio for signaling. **E** When CRMP2 S522D is overexpressed (OE), new ectopic branching zones are created. Because CRMP2 S522D recruits Trio, these new zones are non-permissive and no new branching occurs, although endogenous CRMP2 foci may still permit branching (purple circles). **F** When Trio-Rac1 signaling is lost (Trio LOF), CRMP2 S522D overexpression (OE) creates new branching zones. Because CRMP2 S522D cannot recruit Trio for local signaling, these branching zones are permissive, despite CRMP2 phosphorylation status, and branching increases.

CRMP2 is frequently thought to influence axon patterning through regulating microtubule stability. C-terminal phosphorylation of CRMP2 fully ablates its affinity for microtubules, so pCRMP2 is often considered an inactive form of CRMP2 that exerts control over axon patterning by sudden cessation of microtubule stabilization^30,33,40,62,63^. However, we show that pCRMP2 actively remodels the cytoskeleton through recruitment and activation of Trio. We propose there are regions along the axon where CRMP2 is highly locally enriched, and axon branching may or may not be permitted. Whether or not these CRMP2 foci permit branch formation is regulated by C-terminal CRMP2 phosphorylation, and suppression is performed through Trio GEF1 signaling (Fig. 6). GSK3B-mediated phosphorylation events downstream of CRMP2 S522A (i.e., pT509, pT514, and pS518) may further mediate and regulate the CRMP2-Trio association. Although modulation of CRMP2 S522A reportedly blocks phosphorylation on GSK3B sites, it is possible that modulation of GSK3B sites might further enhance or repress the CRMP2-Trio association. Also, there may be additional CRMP2 modifications that regulate the CRMP2-Trio association. We observe an additional pS522 CRMP2 immunoreactive band in the growth cone fraction, which does not co-immunoprecipitate with Trio. The higher molecular weight of this band and its immunoreactivity for CRMP2 pS522 suggest additional CRMP2 modifications that can repress the CRMP2-Trio association despite phosphorylation on S522.

It is important to note that we do not contend that Trio and CRMP2 are the sole determinants of axon branching. Indeed, it is likely that Trio and CRMP2 interact within the context of a larger protein complex, potentially an adhesome-like structure, which may mediate their interaction and contribute additional regulatory and catalytic elements to limit axon branching. In addition to mediating the Trio-CRMP2 interaction, such an adhesome-like structure may be the ultimate target of Trio-Rac1 signaling, since focal adhesions offer structural stability during axon branching and coordinate the recruitment of intracellular cargo and metabolic components required for axon branch growth. Indeed, the involvement of such a structure would be consistent with the LIMK-cofilin signaling pathway discussed earlier. Further elucidation of these intermediary proteins and signaling partners is essential to a full understanding of the CRMP2-Trio-Rac1 signaling pathway and the mechanism by which it opposes axon branching.

In conclusion, we have elucidated a mechanism of axon branch suppression, mediated by pCRMP2-Trio GEF1 signaling and demonstrated its role in mediating Sema3A signaling. These findings lend complexity to our current understanding of how CRMP2 and Rac1 sculpt the cytoskeleton. Additionally, we show this pathway is not only inhibited by, but inverted by, NDD-associated mutations in Trio-GEF1, positioning Sema3A-pCRMP2-Trio signaling as a potential therapeutic target for Trio-related NDDs.

## Materials and methods

### Animals

The NINDS Animal Care and Use Committee approved all experimental animal use (protocol #1171). Male and female Sprague Dawley outbred rats (Envigo, Cat#: 002) were used at >1 month of age. Rats were housed on a standard 12 hr dark and light cycle. We have complied with all relevant ethical regulations for animal use.

### Experimental plasmid constructs

For Trio knock-down we used short-hairpin RNA against TRIO (shTRIO) or a scrambled sequence (shSCR) under a H1 promoter in an FUGW backbone with GFP under the Ub promoter, as previously described^39^. pcDNA3.1-CRMP2-FLAG was cloned from a CRMP2-V5 construct generously provided by Dr. Yoshio Goshima^33^, and then mutagenized via site-directed mutagenesis to obtain S522A and S522D mutants. shRNA-resistant Trio9S was generated from pCAG-Trio9^39^. Using a Gibson Assembly kit, an HA tag was added to Trio-9S and HA-Trio-9S was subcloned into pcDNA3.1. pcDNA3.1-HA-Trio-9S was mutagenized via site-directed mutagenesis to obtain K1431M, L2051E, and K1431M/L2051E mutants using Agilent’s QuickChange XL site-directed mutagenesis kit. All mutant plasmids were sequence verified by whole plasmid sequencing and have been deposited with Addgene (IDs: 246414; 246415; 246416; 246417; 246418; 246419).

### Antibodies

For immunoprecipitation experiments, a previously described Trio antibody was used^36^. For immunoblotting, we used primary antibodies against Trio (mouse, AbCAM, Cat#: ab194365, RRID: AB_2894973), CRMP2 (mouse, AbCAM, Cat#: Ab62539, RRID:AB_941175), pCRMP2 phosphoS522 (rabbit, ThermoFisher Scientific/Invitrogen, Cat#: pa5-37550, RRID: AB_2554159), FLAG (rabbit, ThermoFisher Scientific/Invitrogen, Cat#: 701629, RRID: AB_2532497) and β-actin (Mouse, Applied Biological Materials Inc., Cat#: G043, RRID:AB_2631287); and HRP-conjugated secondary antibodies against mouse IgG (GE Healthcare/Cytiva; Cat#: NA934, RRID: RRID:AB_772210), rabbit IgG (GE Healthcare/Cytiva; Cat#: NA934, RRID: AB_772206), denatured mouse IgG (Rockland Immunochemicals, Cat# 18-8817-33, RRID:AB_2610851) and denatured rabbit IgG (Rockland Immunochemicals, Cat#: 18-8816-33; RRID:AB_2610848).

For immunofluorescence imaging, we used primary antibodies against GFP (chicken, ThermoFisher Scientific/Invitrogen, Cat#: A10262, RRID: AB_2534023; rabbit, ThermoFisher Scientific/Invitrogen, Cat#: A11122, RRID: AB_221569), FLAG (rabbit, ThermoFisher/Invitrogen, Cat#: 701629, RRID: AB_2532497; rat, AbCAM, Cat#: Ab213519), Tau (guinea pig, Synaptic Systems, Cat#: 314 004, RRID: AB_1547385), HA (rat, Millipore Sigma/Roche, Cat#: 11867423001, RRID:AB_390918), CRMP2 (rabbit, Millipore Sigma/Sigma Aldrich, Cat#: C2993, RRID:AB_1078573) and Trio (mouse, AbCAM, Cat#: ab194365, RRID: AB_2894973). Alexa Fluor-conjugated secondary antibodies were purchased from ThermoFisher Scientific/Invitrogen (Cat#: A-11039, RRID: AB_2534096; Cat#: A-11008, RRID: AB_143165; Cat#: A-21428, RRID: AB_2535849; Cat#: A-21244, RRID: AB_2535812; Cat#: A-10680, RRID: AB_2534062; Cat#: A-21424, RRID: AB_141780; Cat#: A-21235, RRID: AB_2535804; Cat#: A-21450, RRID: AB_2535867).

### Neuronal cultures

Primary hippocampal and cortical neurons were prepared from male and female embryonic day 18 Sprague Dawley rats (Envigo) following the guidelines of the NIH Guide for the Care and Use of Laboratory Animals. The animals were narcotized with CO_2_ and the embryos removed. Embryonic brains were subsequently removed, dissected, and enzymatically and mechanically dissociated prior to plating on poly-D-lysine coated plates and coverslips. Mixed gender cultures were maintained in Neurobasal Medium (Life Technologies, Cat# 21103-049) supplemented with 2% B-27 (Life Technologies, Cat#17504-044) and 2 mM L-Glutamine (Sigma-Aldrich, Cat# G-7513) at 37°C and 5% CO_2_.

### Calcium phosphate transfection

At *DIV 4*, primary cortical cultures were transfected using calcium phosphate transfection (Kwon & Firestein, Methods Mol. Bio, 2013). 15 µg CRMP2-FLAG was incubated at room temperature with 31 µL 2 M CaCl_2_ for 5 minutes in 250 µl H_2_O, then added to 250 µl Hanks Buffered Saline (HBS) and incubated at room temperature for 20 minutes. The DNA-CaCl_2_ mixture was added to *DIV 4* primary cortical cultures in high pH (7.9) neurobasal media and allowed to incubate for 4 hours at 37 °C. Transfected cultures were washed with low pH (7.3) neurobasal media for 30 minutes, then transferred to original culturing media. After 2 days, neurons were collected and lysed for Trio-immunoprecipitation.

### Growth cone fractionation

Growth cone fraction was obtained as described in Pfenniger et al.^64,65^. 6–10 embryonic day 21 or post natal day 2 rat brains were homogenized in 320 mM sucrose buffer containing 20 mM Tris-HCl (pH 7.4), 150 mM NaCl, 1 mM EDTA (pH 8.0) and protease and phosphatase inhibitors and centrifuged at 1000 × *g* for 10 minutes. The supernatant was applied to a 0.75 M sucrose cushion and centrifuged at 242,000 × *g* for 2 hours. The growth cone fraction was extracted from the 0–0.75 M interface with a syringe and solubilized in 1% Triton X-100 lysis buffer containing 50 mM Tris-HCl (pH 7.4), 150 mM NaCl, 1 mM EDTA (pH 8.0) and protease and phosphatase inhibitors (cOmplete Protease Inhibitor Cocktail, Millipore Sigma/Roche, Cat#: 11697498001; Phosphatase Inhibitor Cocktail 2, Millipore Sigma/Sigma Aldrich, Cat#: P5726; Phosphatase Inhibitor Cocktail 3, Millipore Sigma/Sigma Aldrich, Cat#: P0044).

### Immunoprecipitation and immunoblotting

For immunoprecipitation of endogenous Trio, 100 μg of Trio antibody^36^ was incubated with lysates overnight at 4 °C while rocking. The following day, Protein A-agarose beads (Millipore Sigma/Sigma Aldrich, Cat#: P6649-1ML) were added to the samples and allowed to incubate for 4 hours at 4 °C while rocking. Beads were washed 3 times with 1% Triton X-100 lysis buffer (described above) and denatured for 5 minutes at 95 °C. Immunoprecipitates were either analyzed by mass spectrometry or immunoblotting. For immunoblotting, immunoprecipitates were separated via SDS-PAGE on a 7% acrylamide gel, then transferred to PVDF membrane and immunoblotted. Blots were developed using Pierce ECL Western Blotting Substrate (Thermo, 32109) and BioRAD Chemidoc imaging system.

### Dephosphorylation assay

Trio immunoprecipitation was performed as described above, in absence of EDTA and phosphatase inhibitors (cOmplete EDTA-free Protease Inhibitor cocktail, Millipore Sigma/Roche, Cat#: 11873580001). On-bead dephosphorylation was performed on Trio-immunoprecipitates after the washing step. 60 units of CIAP (Takara, 2250 A) were added to Trio-immunoprecipitates in 100 μL manufacturer supplied buffer and allowed to incubate at 4 C for 1 hour.

### Mass spectrometry analysis

Immunoprecipitated samples were prepared as described in immunoprecipitation and immunoblotting. Samples were resolved by SDS-PAGE. The gel bands were excised and washed overnight in 50% methanol with 10% acetic acid. Proteins were reduced with 5 mM Tris(2-carboxyethyl)phosphine hydrochloride at room temperature for 1 hr, alkylated with 5 mM N-ethylmaleimide for 10 min, and digested with trypsin (Promega) 1:10 (w/w) at 37 °C for 18 hr. Tryptic digests were extracted from the gel and cleaned with an Oasis HLB 30 mg plate (Waters). Peptides were separated on an ES902 column. Mobile phase A contains 0.1% formic acid in LC-MS grade water, and Mobile phase B contains 0.1% formic acid in LC-MS grade acetonitrile. Mobile phase B was increased from 3% to 18% in 63 min, then from 18% to 30% in 9 min. LC-MS/MS data were acquired in data-dependent mode. The MS1 scans were performed in orbitrap with a resolution of 120 K, a mass range of 375–1500 m/z, and an AGC target of 4 ×10^5^. The quadrupole isolation window is 2 m/z. The precursor ion intensity threshold to trigger the MS/MS scan was set at 1 ×10^4^. MS2 scans were conducted in the ion trap. Peptides were fragmented with the HCD method and the collision energy was fixed at 35%. MS1 scan was performed every 3 sec. As many MS2 scans were acquired within the 3-sec cycle.

Proteome Discoverer software version 2.4 was used for protein identification and quantitation. Raw data were searched against the Sprot Rat database and the house-built database containing the sequence of IgG used in the pull-down experiment. Up to 1 missed cleavage was allowed for trypsin digestion. NEM on cysteines was set as a fixed modification. Variable modifications include Oxidation (M), Met-loss (Protein-N-term), and Acetyl (Protein N-term). Mass tolerances for MS1 and MS2 scans were set to 5 ppm and 0.5 Da, respectively. A percolator was used for PSM validation. The search results were filtered by a false discovery rate of 1% at the protein level. Protein abundance values were calculated for all proteins identified by summing the abundance of unique peptides matched to that protein. The normalization was performed against IgG amount. Protein ratios were calculated by comparing the protein abundances between 2 conditions. p-values were calculated with the ANOVA method. Proteins matched with 1 peptide were filtered out.

### Imaging

Hippocampal neurons were co-transfected with shRNA-GFP, SNAP or FLAG-CRMP2, and/or HA-Trio9S using Lipofectamine 2000 (ThermoFisher Scientific/Invitrogen, Cat#: 11668019) on *DIV 3*. For Sema3A-branching experiments, neurons were treated with 700 ng/mL Semaphorin3A (Acrobiosystems; Cat#: SEA-H5259-100ug) at *DIV4*. On *DIV 7*, Neurons were fixed in 4% paraformaldehyde/4% sucrose and stained against GFP, FLAG, HA, Tau, and/or F-actin (Phalloidin Alexa Fluor 647, ThermoFisher Scientific/Invitrogen, Cat#: A22287). Coverslips were mounted with Prolong Gold Antifade mountant (ThermoFisher Scientific/Invitrogen, Cat#: P36930) and imaged on either a Zeiss LSM 800 confocal microscope or a Zeiss Axioimager.M2 with apotome. For live cell imaging, neurons were plated on glass-bottom dishes, labeled with SNAP-Cell TMR-STAR (New England Biolabs, S9105), and imaged in phenol red-free neurobasal media (ThermoFisher Scientific/Gibco, 12348017) over 6 minutes on a Zeiss LSM 880 confocal microscope with an incubator stage insert set at 37°C and 5% CO_2_. For image analysis, Carl Zeiss Image files (.czi) were loaded into FIJI using the Bio-Formats Importer^66,67^. For branch length and density measurements, the Simple Neurite Tracer FIJI plug-in was used to manually trace the GFP/488 channel of each image, and then used to measure branch length, number, order, and the longest shortest path (longest possible axon)^41^. Colocalization was measured using the Coloc 2 FIJI plug-in (https://imagej.net/plugins/coloc-2) limiting our analyses to an axonal ROI by using the GFP channel to create an axon-specific mask. Additionally, we used bisection thresholding to limit our analyses to non-zero pixels. To measure pPAK signal, we created a mask of pPAK puncta by smoothing and thresholding the pPAK signal. Then, we combined this mask with a mask defined by the axonal GFP signal to create a mask of pPAK puncta belonging to the transfected axon. From this combined mask, we created ROIs, which we used to analyze the raw (i.e. non-smoothed and non-thresholded) pPAK signal and thus exclude contribution of background immunostaining and pPAK signaling falling outside of the transfected neuron. pPAK intensity was calculated by measuring the total amount of pPAK fluorescence intensity and then normalizing to ROI size. All measurements were made prior to any image brightness and contrast adjustments to representative images.

### Statistics and reproducibility

Data were tabulated in Microsoft Excel and then transferred to GraphPad Prism Version 9.4.1 for statistical analyses. Details of statistical tests are included in the text and figure legends and in the source data supplementary file. Generally, Two-way ANOVA was used to analyze data sets with more than one experimental variable; one-way ANOVA was used to analyze data sets with one experimental variable but more than two comparison groups; and t-tests were used to analyze data sets with one experimental variable but only two comparison groups. When possible, data were collected and analyzed blind to condition.

### Reporting summary

Further information on research design is available in the Nature Portfolio Reporting Summary linked to this article.

## Supplementary information

Supplemental Information

Description of Additional Supplementary Files

Supplementary Data 1

Supplementary Data 2

Reporting summary

## Data Availability

The data that support the findings of this study are available within the paper and its supplementary information files (Supplementary Data 1 and Supplementary Data 2). Additionally, mass spectrometric data have been deposited with proteomeXchange (dataset identifier: PXD067662).
