# Supplementary material for: Trio and CRMP2 regulate axon branching and Semaphorin3A signaling
Source: Commun Biol. 2025 Nov 25;8:1662. doi: 10.1038/s42003-025-08988-8 (PMC12647243; doi:10.1038/s42003-025-08988-8)

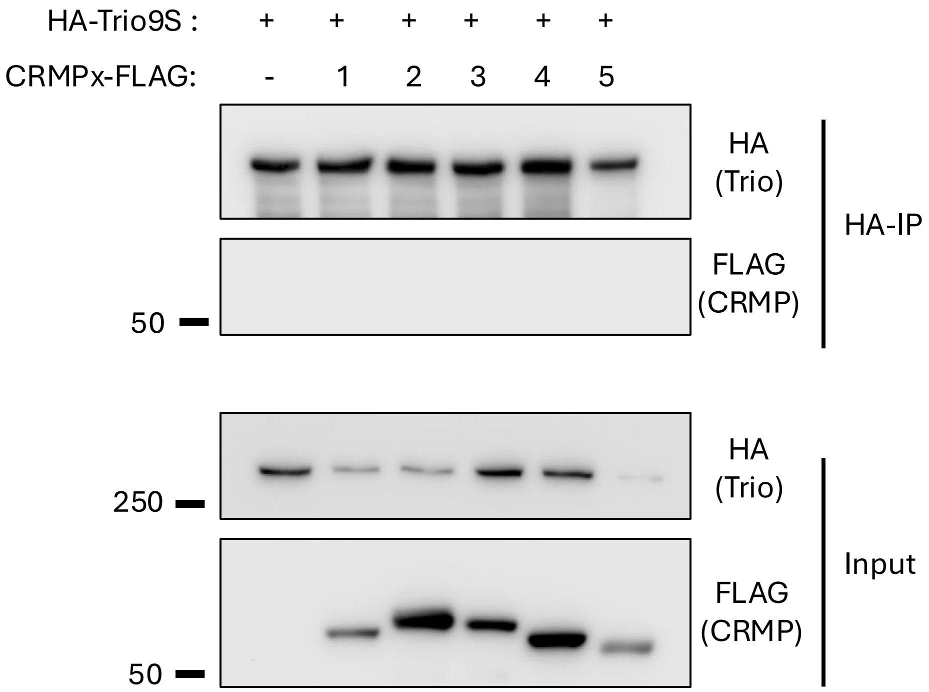

Figure S1: Trio and CRMP2 do not interact in HEK cells. HEK293T cells were transfected with FLAG-tagged CRMP constructs and HA-tagged Trio9S. HEK cells were lysed and HA-Trio9S was immunoprecipitated using HA antibody, then subjected to western blot. Representative of 3 experimental replicates

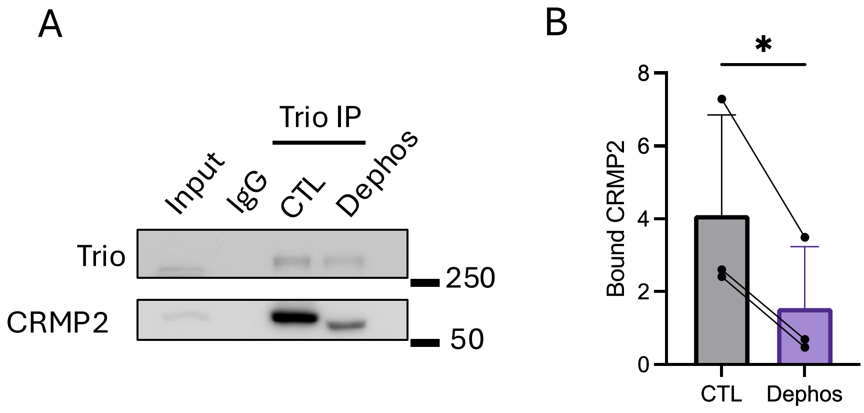

Figure S2: The Trio/CRMP2 interaction is enhanced by CRMP2 phosphorylation. A)Immunoprecipitation of Trio with on-bead dephosphorylation with 60 units of Calf Intestinal Phosphatase. (N = 3 experimental replicates) B) Quantification of A). Ratio paired two-sided t-test was performed. $p = 0.0430$

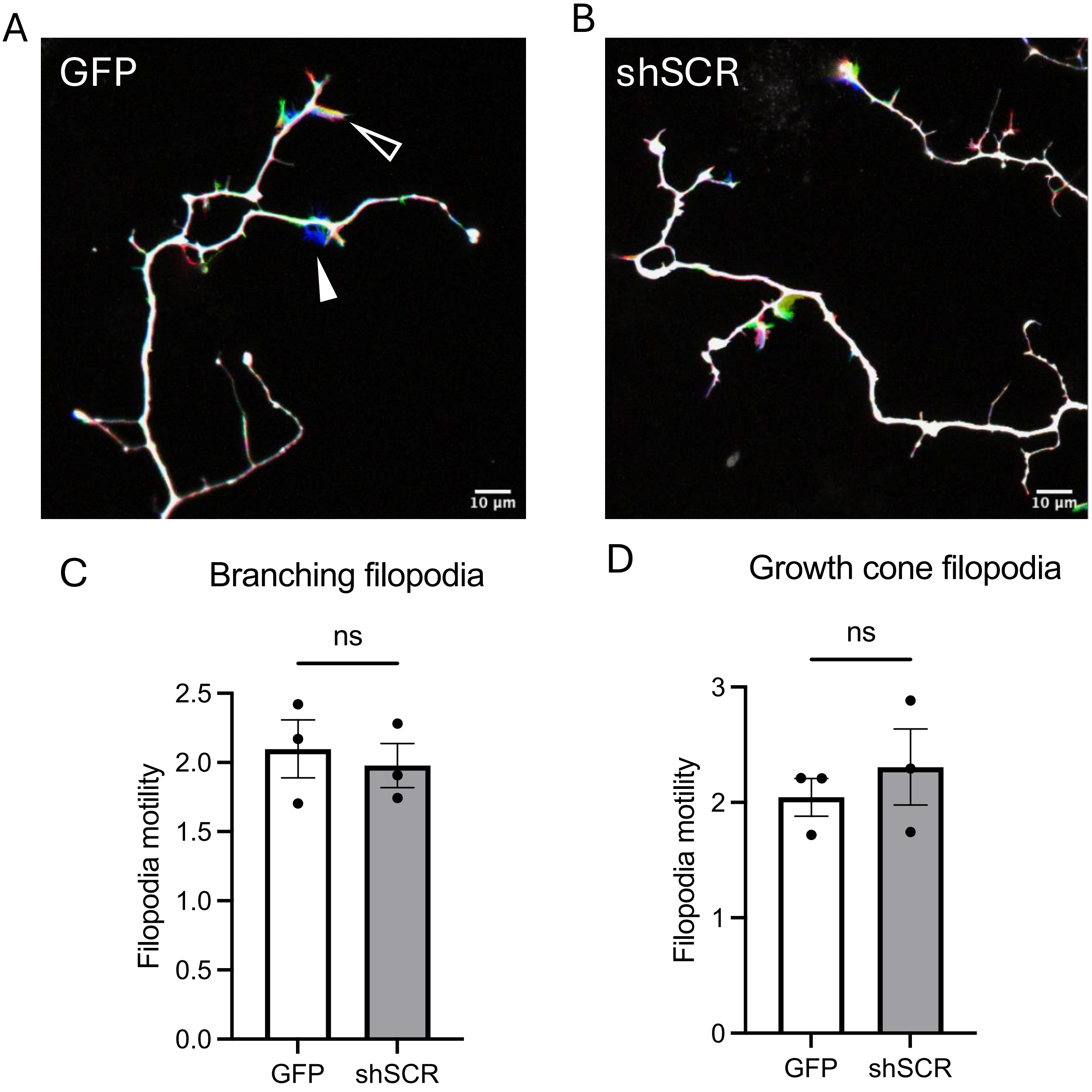

Fig. S3: Expression of shSCR does not significantly alter filopodial motility compared to GFP. A) DIV 7 neuron transfected with GFP, imaged over 6 minutes, and temporally color-coded B) DIV 7 neuron transfected with shSCR-GFP, imaged over 6 minutes, and temporally color-coded . C) No difference in motility of branching filopodia (filled arrow) was observed (N = 3 experimental replicates, n = 5-7 axon compartments, p = 0.6712, unpaired two-sided t-test) D) No difference in motility of growth cone filopodia (empty arrow) was observed (N = 3 experimental replicates, n = 5-7 axon compartments, p = 0.5175, unpaired two-sided t-test)

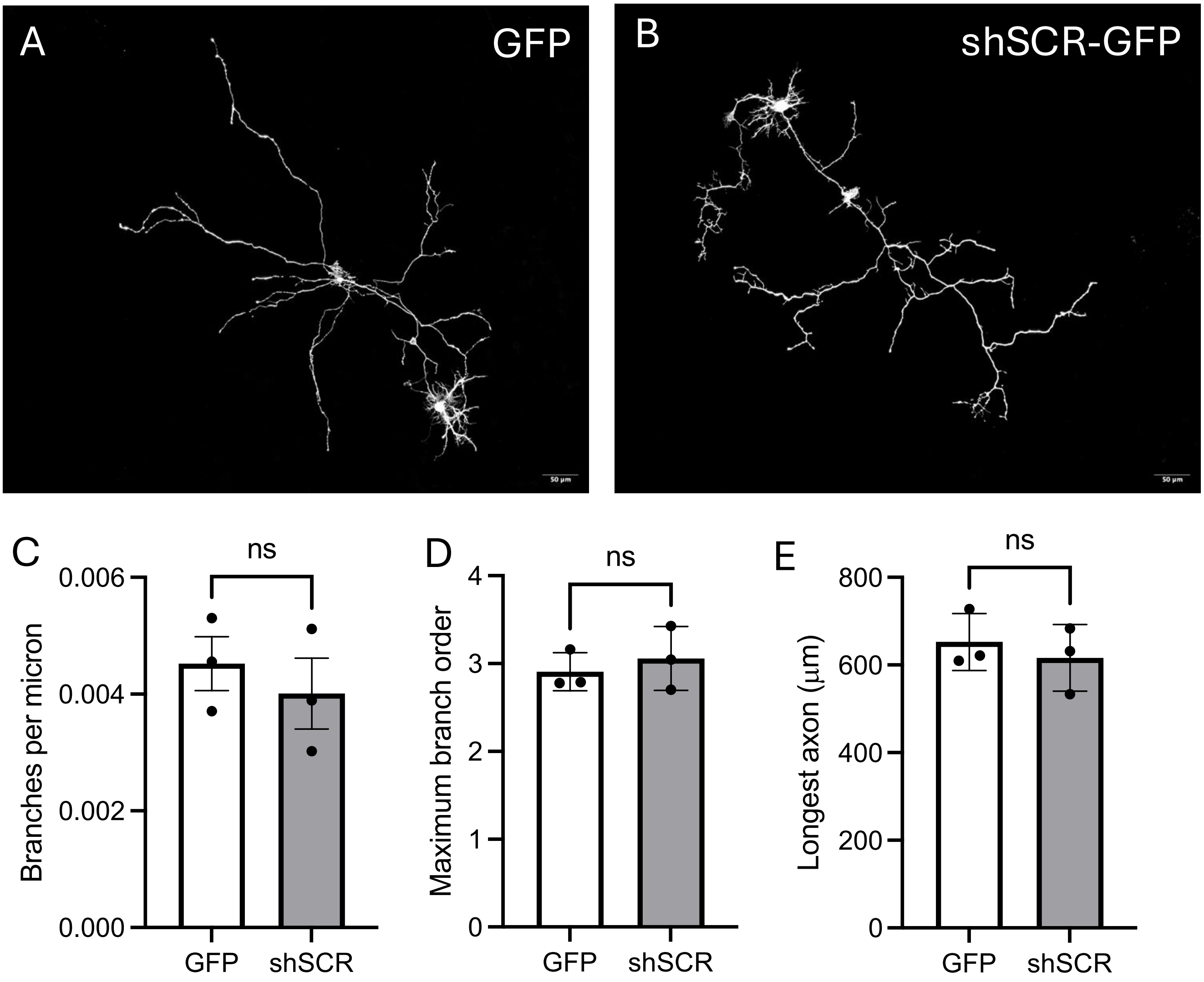

Fig. S4: Expression of shSCR does not significantly alter axon morphology compared to GFP. A) DIV 7 neuron transfected with GFP and stained against GFP (shown) and Tau B) DIV 7 neuron transfected with shSCR-GFP and stained against GFP (shown) and Tau. Tau positive axon branches in A and B were traced using simple neurite tracer and analyzed. C) No difference in branches per micron was observed (N = 3 experimental replicates, n = 7-10 neurons, $p = 0.5390$, unpaired two-sided t-test) D) No difference in maximum branch order was observed (N = 3 experimental replicates, n = 7-10 neurons, $p = 0.5746$, unpaired two-sided t-test) E) No difference in the length of the longest axon was observed (N = 3 experimental replicates, n = 7-10 neurons, $p = 0.5599$, unpaired two-sided t-test)

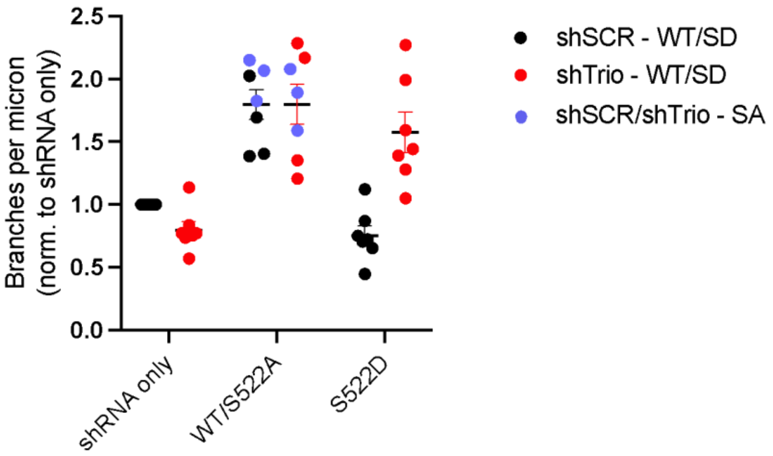

Fig. S5: CRMP2 S522A overexpression phenocopies WT CRMP2 overexpression.
Neurons were transfected as described in Fig.3. For 4 preliminary experimental replicates, WT CRMP2 was used in lieu of CRMP2 S522A. CRMP2 S522A is shown here in periwinkle, overlaying the CRMP2 WT data in black (shSCR) versus red (shTrio). (N = 3-4 experimental replicates, n = 7-10 neurons)

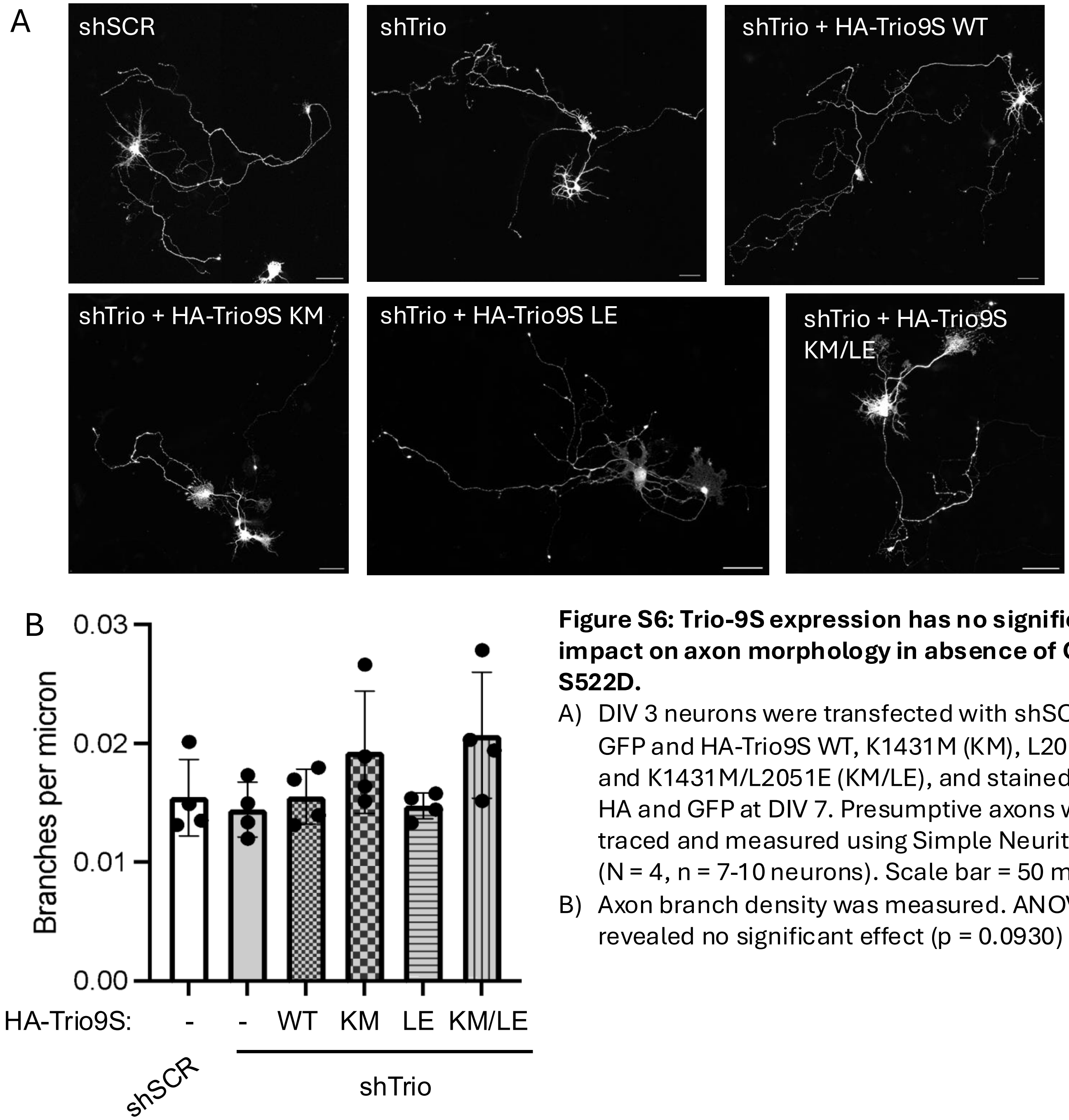

Figure S6: Trio-9S expression has no significant impact on axon morphology in absence of CRMP2-S522D.

A) DIV 3 neurons were transfected with shSCR/shTrio-GFP and HA-Trio9S WT, K1431M (KM), L2051E (LE) and K1431M/L2051E (KM/LE), and stained against HA and GFP at DIV 7. Presumptive axons were traced and measured using Simple Neurite Tracer. (N = 4, n = 7-10 neurons). Scale bar = 50 microns

B) Axon branch density was measured. ANOVA revealed no significant effect ($p = 0.0930$)

Uncropped blots (Figure 1)

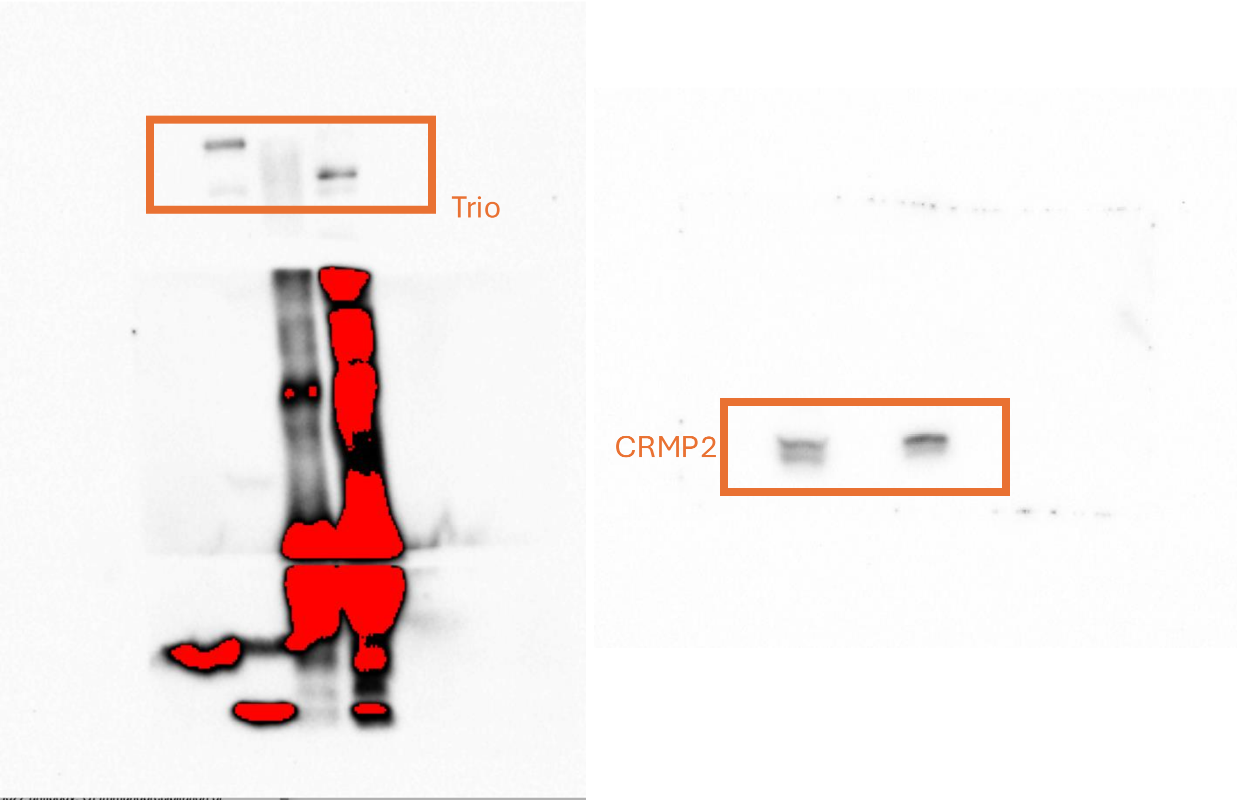

Figure 1C

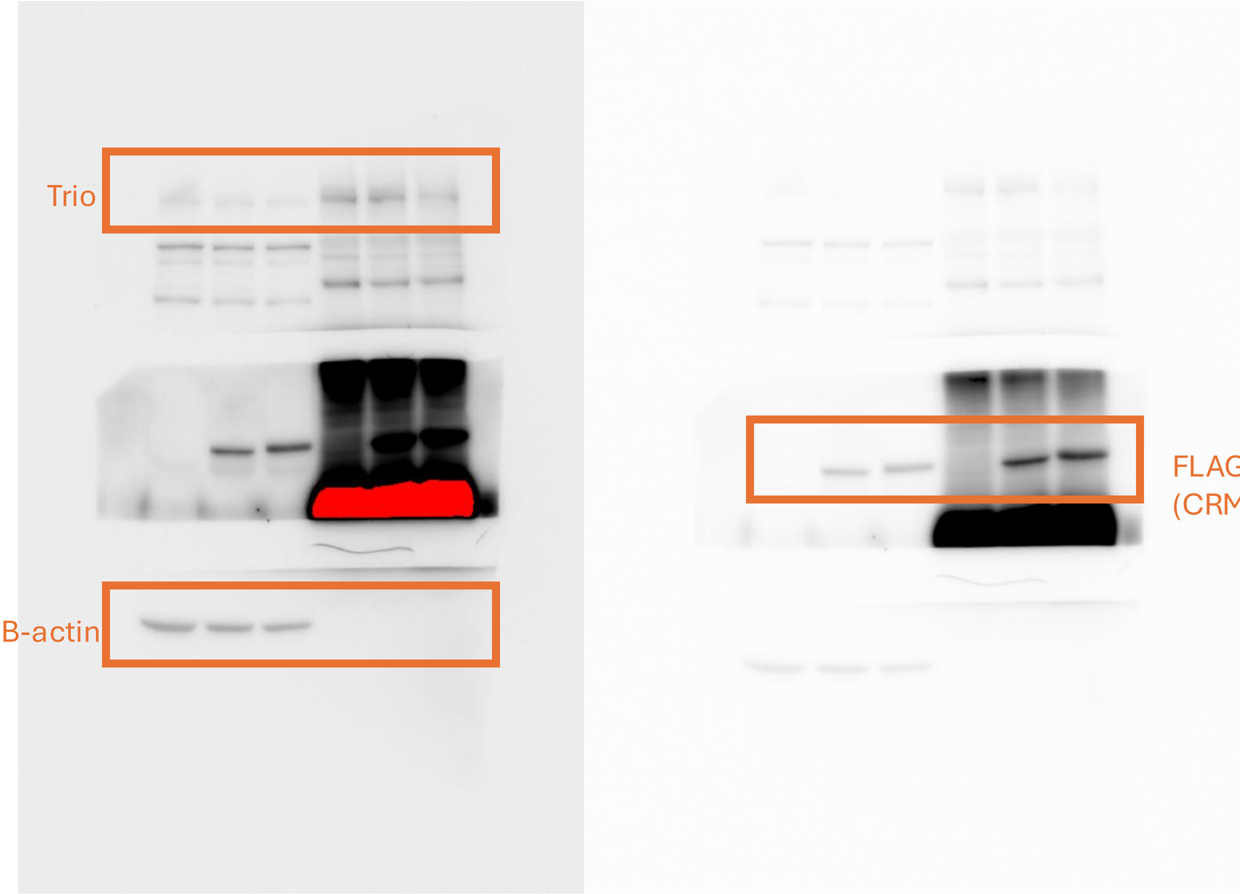

Figure 1G

Uncropped blots (Figure S1)

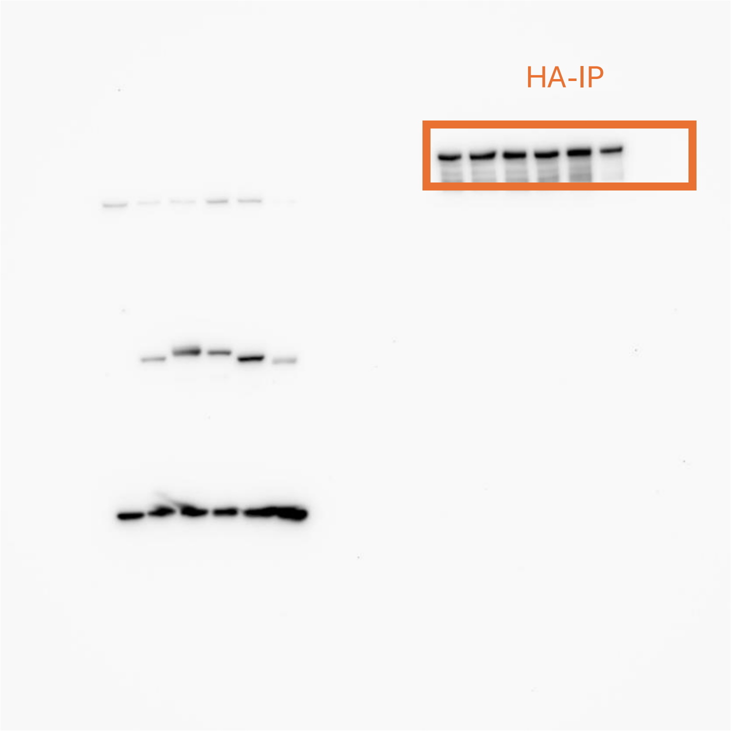

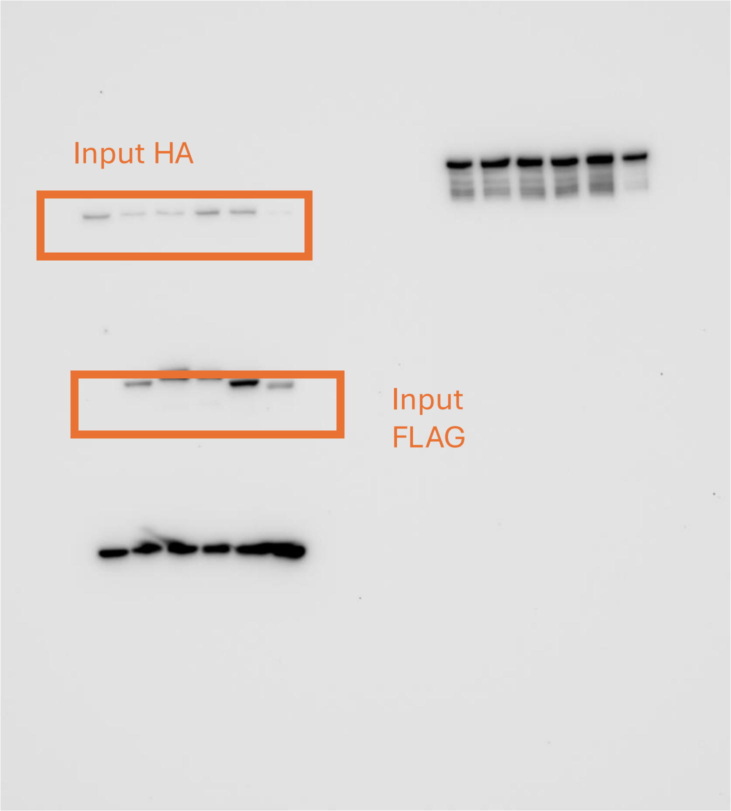

Uncropped blots (Figure S2)

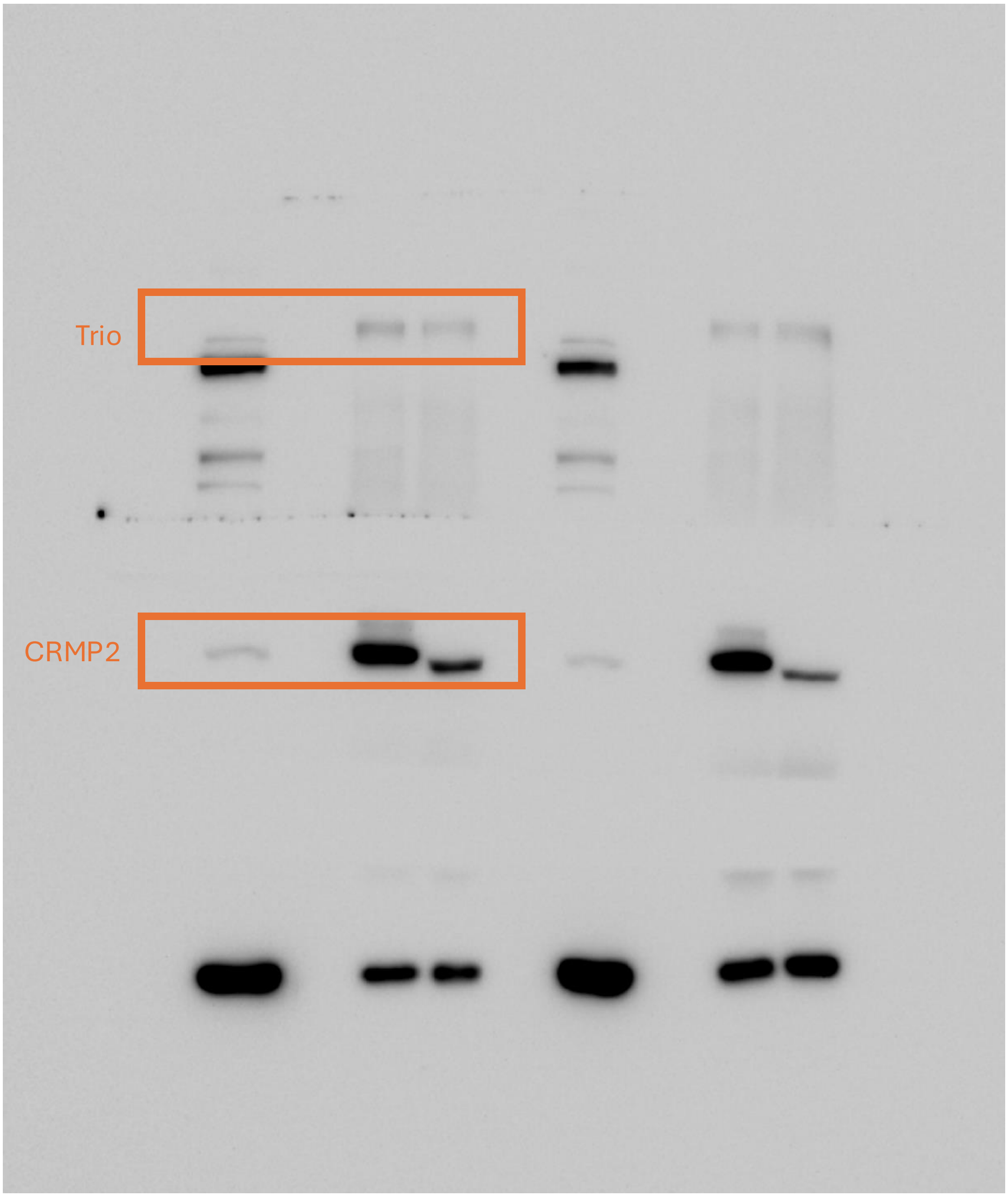

Supplement: Supplementary file 1 — Supplemental Information [file 42003_2025_8988_MOESM1_ESM.pdf]
