# Supplementary material for: Trio and CRMP2 regulate axon branching and Semaphorin3A signaling
Source: Commun Biol. 2025 Nov 25;8:1662. doi: 10.1038/s42003-025-08988-8 (PMC12647243; doi:10.1038/s42003-025-08988-8)
Supplement: Supplementary file 2 — Description of Additional Supplementary Files [file 42003_2025_8988_MOESM2_ESM.pdf]

Description of Additional Supplementary Files

File name: Supplementary Data 1

Description: Mass Spectrometry Data

File name: Supplementary Data 2

Description: Source Data
